# Contextual Modulation of Semantic Coherence in vmPFC Patients’ Mental Constructions

**DOI:** 10.3390/e28050488

**Published:** 2026-04-24

**Authors:** Debora Stendardi, Matteo Reale, Francesca Dalle Piagge, Elena Garavini, Michela Grasselli, Elisa Ciaramelli

**Affiliations:** 1 Dipartimento di Psicologia ‘Renzo Canestrari’, University of Bologna, 40126 Bologna, Italy; elisa.ciaramelli@unibo.it; 2 Centro di Studi e Ricerche in Neuroscienze Cognitive, University of Bologna, Cesena Campus, 47521 Cesena, Italy; matteo.reale@studio.unibo.it (M.R.); francesc.dallepiagge@studio.unibo.it (F.D.P.); elena.garavini@studio.unibo.it (E.G.); michela.grasselli@studio.unibo.it (M.G.)

**Keywords:** ventromedial prefrontal cortex, schema-based cognition, semantic coherence

## Abstract

Previous evidence has identified the ventromedial prefrontal cortex (vmPFC) as crucial for implementing high-level semantic memory structures (schemas) during event construction. If this is the case, one would expect reduced semantic coherence in events mentally constructed by vmPFC patients compared to healthy and brain-damaged controls. We tested this prediction by having participants mentally construct events using objects as cues and reanalyzing a published dataset using sentences as cues. In both cases, we measured the semantic coherence of patients’ mental constructions and their semantic coherence with the cue, using transformer-based sentence embeddings (S-BERT), and further corroborated the findings with E5 Multilingual and E5 Italian embedding models. Our results reveal that the hypothesized impairment in semantic coherence following vmPFC damage is, in fact, task-dependent. With minimal (object) cues, vmPFC patients’ reports exhibited reduced local coherence, increased connectedness to the cues, and reduced lexical diversity. In contrast, with extended (sentence) cues, they showed preserved- or even enhanced-local and global coherence. We suggest that vmPFC integrity is necessary to trigger schema activation under minimal cue conditions. Although extended cues may facilitate schema activation, schemas are degraded and essentialized following vmPFC damage, thereby constraining patients’ mental constructions within a narrower—hence overly coherent—semantic space.

## 1. Introduction

Future events do not exist until they are constructed, yet humans routinely produce them with remarkable ease. What is it that allows us such a capacity?

Constructing events requires actively selecting, organizing, and integrating information into a coherent structure. Indeed, future events follow a thread, a continuity that gives rise to a narrative sequence. This narrative sequence can be more or less constrained, i.e., it can follow (or diverge from) an event script. Event scripts are a particular type of schema, namely, an “associative network structure, based on multiple episodes, and lacking unit detail” [1]. Event scripts provide the building blocks for constructing events, shaping their internal structure and temporal flow [2,3,4]. Previous research has reliably implicated the ventromedial prefrontal cortex (vmPFC) in both schema-based cognition and event construction [4,5,6,7,8]. Recently, Bein & Niv [9] proposed that the mPFC supports both schematic cognition and the orchestration of memory reactivation through interactions with posterior brain regions. mPFC is in fact sensitive to the structure of specific schemas during event perception [10,11], and is consistently activated during autobiographical memory retrieval and episodic future thinking [12,13,14]. Further, prefrontal lesions impair sequencing and boundary detection in scripts [15,16], and vmPFC lesions hinder the association of actions with their relevant schemas, especially in confabulating patients [17,18,19,20].

Schemas act as the scaffolding structure supporting event construction [2]: according to a recent theory [21], during event construction, vmPFC initiates the activation of schema-based knowledge in neocortex, which is used by the hippocampus to construct a sketchy scene (see also [3,4]). Then, a hippocampal–vmPFC interplay would mediate schema-based retrieval to monitor the unfolding of a complex event (see also [22]). This perspective aligns with evidence showing heightened recruitment of the vmPFC–hippocampal axis during rule transfer, supporting schema reinstatement [23]. Recent 7T fMRI evidence further underscores vmPFC’s role in constructing temporally extended scenarios beyond single scenes, and in integrating elements within events and narratives [24]. Ali et al. [25] used single cue words (e.g., “hope”) and instructed participants to activate one of three distinct representations (scenarios, objects, or semantic definitions). The vmPFC was the only region in which the classifier accuracy with the Multi-Voxel Pattern Analysis for scenarios exceeded that of object construction and semantic definitions, highlighting its role as a critical hub that integrates low- and high-level mental representations. Indeed, vmPFC patients typically produce impoverished accounts during event construction [5,6,7,26,27]. Drawing from this, we have previously found that such impairment may stem from a degradation of the schemas that are necessary for event construction, which become broad, or “nebulous,” following vmPFC damage ([28]; see also [17,18,29]). As such, when specifically probed to report the actions that compose an event script, vmPFC patients retain its “backbone”, namely, the actions that are more central to it (major actions, see [28]). Therefore, our (and others’) previous results suggest that vmPFC patients do indeed use event schemas, but in a degraded form ([4,17,28,30]).

In a recent study, Park and colleagues [31] had participants watch a temporally scrambled movie and later recount its inferred story. The authors used semantic similarity to assess content encoding and event sequencing abilities. Crucially, functional connectivity between the hippocampus and vmPFC predicted sequencing performance during moments where integration between past and present information was required, and these moments were identified by means of pre-set event narratives and language models. Hence, the authors proposed a role of the hippocampal–vmPFC axis for the integration of events into coherent narratives (i.e., scripts). Importantly, this suggests that the semantic coherence of text is influenced by whether a narrative conforms to an event script (or not).

Building on this, here, we ask whether impoverished schematic knowledge could influence the structure of discourse and the language used during event construction in patients with vmPFC damage. Kurczek & Duff [32] investigated the effect of vmPFC lesions on cohesion and coherence in spoken discourse and found them preserved in vmPFC patients. However, if schematic knowledge is degraded after a vmPFC lesion, and if stories that follow an event script are more semantically coherent, we should expect vmPFC patients to display a lower semantic coherence of discourse during event construction. Computational approaches allow researchers to formally quantify semantic coherence in discourse. In particular, sentence-level embedding models can capture the trajectory of meaning across a narrative and have been successfully employed to quantify discourse alterations in clinical populations such as schizophrenia and Alzheimer’s disease [33,34].

Here, we use sentence-level embedding models to examine the semantic coherence of discourse (both at the local and at the global level) in vmPFC patients in two experiments, using objects or sentences as cues for event construction (see [28]). Furthermore, we explore whether vmPFC patients typically produce sentences with a different grammatical structure (e.g., use of nouns, adjectives, etc.), their lexical richness (unique word usage), and whether these could be influenced by the use of minimal (i.e., objects) or extended (i.e., sentences) cues.

## 2. Experiment 1: Object Cues

### 2.1. Methods

#### 2.1.1. Participants

**Figure 1:**
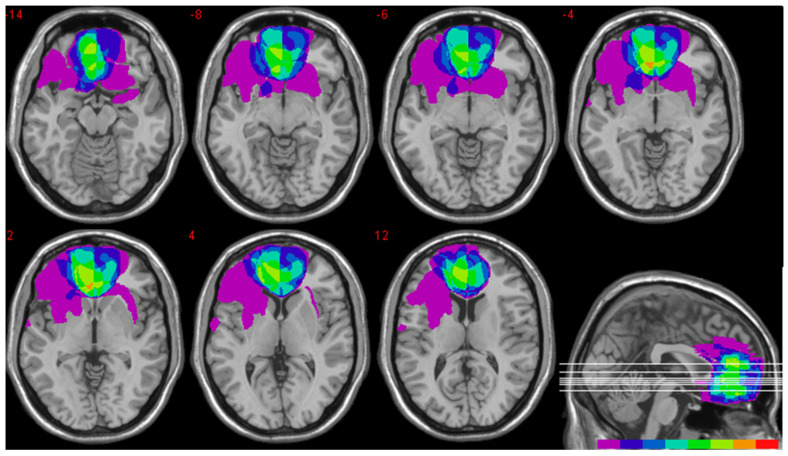
Location and overlap of vmPFC patients’ brain lesions. Lesions are projected on the same axial slices and on the mesial view of the standard Montreal Neurological Institute brain. The level of the axial slices is indicated by white horizontal lines on the mesial view of the brain. Numbers in red indicate z-coordinates. The color bar indicates the number of overlapping lesions.

A total of 52 healthy individuals (29 M, mean age = 58.5 ± 2.6 years; mean education = 12 ± 3.4 years), 8 patients with vmPFC lesions (5 M, mean age = 59.8 ± 7.3 years; mean education = 10.1 ± 3.6 years; see Figure 1 for the lesion overlap), and 8 patients with lesions not encroaching vmPFC (6 M, mean age = 59.5 ± 16.2; mean education = 12.3 ± 5.3) took part in the study. Participant groups were matched in age (BF_10_ = 0.2), education (BF_10_ = 0.4), and sex ratio (BF_10_ = 0.2). All patients were in a stable chronic phase (>3 months post-injury). Control patients had lesions including the occipital cortex (two right, one left), the temporal cortex (two right, one left), the right fronto-insular formation (one patient), and the mesencephalon (one patient). Participants provided informed consent to participate in the study, which was approved by the ethical committees of the University of Bologna and the Regional Health Service of Emilia Romagna.

All vmPFC patients showed preserved short-term memory (digit span performance, Equivalent Scores ≥ 1: mean = 3.5 ± 0.8; [35]). Long-term memory was impaired in three vmPFC patients on the prose passage recall task (Equivalent Score < 1; see [35]; mean ES = 1.6 ± 1.5). Executive functioning was assessed with the Weigl Card Sorting Test for one patient (who performed within the normal range, Equivalent Score = 2; see [36]) and with the Wisconsin Card Sorting Test for the other seven patients, six of whom displayed an impaired performance (Standard Score ≤ 81, mean = 71.7 ± 11.7; see [37]).

#### 2.1.2. Procedure

Participants were shown a set of twelve objects (taken from [38]), one at a time, on a white background on a computer screen. Their task was to first imagine a scene surrounding the object, which remained on screen until participants successfully reported having a scene in mind. Then, they had to describe the scene and an event that might take place in it. Participants were asked to imagine and describe the scene and the event out loud, in as much detail as possible. Specifically, the 12 objects were: a whirlpool tub, a bench, a swing, a gas pump, a pen holder, a shopping trolley, a balloon, a feather, a candy, a rearview mirror, a fishing rod, and a graduation cap. All reports were audio-recorded and transcribed verbatim.

#### 2.1.3. Semantic Analyses

For both datasets, we adopted a data-driven approach in which we characterized the semantic properties of vmPFC patients’ discourse and how it relates semantically to its cue (i.e., the object or sentence). For this reason, we adopt a sentence-embedding approach based on Sentence-BERT (S-BERT), a transformer model designed to generate context-sensitive vector representations of text. S-BERT is a modification of the popular BERT (Bidirectional Encoder Representations from Transformers) model. Unlike BERT, S-BERT is trained to produce sentence embeddings (not token, i.e., word embeddings) that are semantically meaningful; hence, sentences that have similar meanings should have similar vector representations (embeddings) in the vector space ([39]). S-BERT uses siamese or triplet networks during training to directly output meaningful vectors fixed in dimension size (768), optimizing the computation of cosine similarity for pairwise comparisons. Therefore, after fine-tuning, S-BERT can take a sentence and output a fixed-size vector (embedding) in a single forward pass. Here, we use a pre-trained and fine-tuned S-BERT model (nickprock/sentence-bert-base-italian-uncased), specifically designed for Italian language text.

For *local coherence* analyses, we compute sentence-to-sentence cosine similarities across the discourse for all adjacent sentence pairs, deriving mean similarity (overall flow), minimum (outlier sentences or topic drifts), and maximum (peak alignments to the previous sentence). Narratives were segmented into sentences using spaCy’s Italian sentencizer, which identifies sentence boundaries based on punctuation marks and Italian-specific linguistic rules. Text preprocessing was kept to a minimum, leveraging the intrinsic capabilities of the S-BERT model. Therefore, lemmatization and stopword removal were omitted. These steps are not only unnecessary for transformer-based models like S-BERT, but may also degrade performance [40,41], as S-BERT employs contextual embeddings that already account for morphological variation and for the functional role of stopwords within sentences. During the embedding generation phase, all sentence embeddings were subjected to L2 (Euclidean) normalization. This procedure scales the vectors to have a unit length (magnitude of 1), which is a standard practice that improves the reliability of the subsequent cosine similarity calculations [42]. To corroborate our findings, we performed the same analyses using the E5-large multilingual (intfloat/multilingual-e5-large; [43,44]) and the E5-multilingual large Italian (mik3ml/multilingual-e5-large-ita) models (see Supplementary Materials).

**Figure 2:**
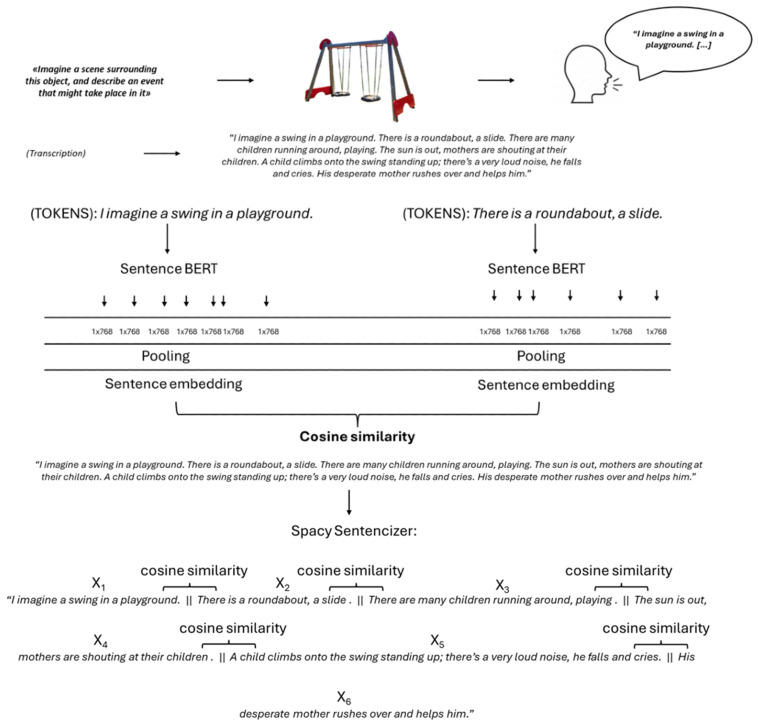
Experimental procedure and semantic analyses.

In both studies, the average semantic coherence is obtained as the mean of the semantic similarities of all adjacent sentences, thus yielding a measure of average local coherence of the text. The minimal/maximal point of coherence is the smallest/largest value of coherence between adjacent sentences in the text. This procedure is elucidated in Figure 2. To compute *global semantic coherence*, sentence embeddings were normalized and averaged to obtain a centroid vector representing the overall semantic content of the text. Then, we calculated the cosine similarity between each sentence embedding and the centroid. The average of these values was used as an index of global coherence, with higher values indicating greater semantic consistency across the narrative. Finally, to characterize stories’ anchoring to their cue, we computed the text-to-title average cosine similarity in the dense vector space (for the object dataset, the title is the object’s name; for the event dataset, it is the cue sentence).

Because measures (average, minimal, and maximal) of semantic similarity can be influenced by the length of a report, and because our vmPFC patients’ utterances contained, on average, fewer sentences than healthy participants in both datasets (Exp. 1: BF_10_ = 3.3; mean vmPFC = 2.6; healthy controls = 4.2; control patients = 2.8; Exp. 2: BF_10_ = 14.6; mean vmPFC = 3.1; healthy controls = 7.7; control patients = 2.8), across analyses we first checked whether semantic similarity measures correlate with the length of stories (number of sentences), and, if so, we statistically control for it by inputting the variable length as a covariate in the Bayesian model (dependent variable: semantic coherence measure; independent variable: group). In addition, only reports including at least 3 sentences were considered in these analyses. This caused the exclusion of one vmPFC patient and two control patients in Exp. 1 and four vmPFC patients and four control patients in Exp. 2. Even after the exclusions, in both Exp. 1 (age: vmPFC = 58 ± 5.8; healthy controls = 58.5 ± 2.6; control patients = 57.2 ± 17.8; BF_10_ = 0.2; education: vmPFC = 10.7 ± 3.4; healthy controls = 12.2 ± 3.4; control patients = 14.2 ± 4.6; BF_10_ = 0.5; sex: vmPFC = 5 M; healthy controls = 29 M; control patients = 4 M, BF_10_ = 0.3) and Exp. 2 (age: vmPFC = 57.2 ± 7; healthy controls = 58.1 ± 6.5; control patients = 56.3 ± 15.6; BF_10_ = 0.3; education: vmPFC = 10.2 ± 3.8; healthy controls = 11.2 ± 3.7; control patients = 12.6 ± 3.1; BF_10_ = 0.3; sex: vmPFC = 2 M; healthy controls = 32 M; control patients = 7 M, BF_10_ = 1.2), participants groups were matched for age, education, and sex ratio.

#### 2.1.4. Linguistic Analyses

To characterize the linguistic aspects of participants’ narratives, each sentence was tokenized and annotated with part-of-speech (POS) tags using the Italian spaCy model (spaCy version 3.8.7; it_core_news_md version 3.8.0). POS tagging is a procedure that assigns to each word its corresponding grammatical function in a sentence (e.g., Verbs, Pronouns), and counts how often they appear in a narrative. Only alphabetic tokens were retained for POS analysis. For each text, the relative frequency of each POS category was calculated as a percentage of the total alphabetic tokens (words) to ensure normalization for length and hence meaningful comparisons among groups. POS categories were Auxiliary Verb, Adposition, Determiner, Noun, Adverb, Pronoun, Verb, Subordinating Conjunction, Coordinating Conjunction, Adjective, Proper Noun, Interjection, Other/Unknown.

Furthermore, to explore whether vmPFC patients show an overall impoverished lexicon, we also computed the moving average type-token ratio (MATTR; [45]). MATTR is an extension of the average type-token ratio (TTR), which is simply the proportion of unique tokens (words) in a text divided by the total number of words. Instead, MATTR is given by the average type-token ratio (TTR) computed over a sliding window across the text, thus yielding an index of lexical diversity that is insensitive to text length [46]. Here, we used a sliding window of size 25 tokens but also corroborated the findings by additionally adopting a 15-token window.

All semantic and linguistic analyses, besides MATTR (S-BERT coherence measures, POS tagging), are performed in Python (version 3.13.2): MATTR scores are computed in RStudio, (version 2022.2.0.443) along with all statistical analyses (R version 4.4.1). For statistical analyses, we compute the Bayes Factor of Bayesian ANOVAs (or regressions) from the package BayesFactor (version 0.9.12.4.7) using the functions anovaBF and generalTestBF, always using the default prior settings and the default number of MCMC iterations (see [47]). Post-hoc comparisons are obtained from the bain package (version 0.2.11), using the BF.c, which denotes the Bayes factor of the hypothesis at hand versus its complement (see [48]). The complete dataset (excluding narratives, which we do not share for privacy concerns) and the scripts for semantic coherence computation and all statistical analyses are available on the OSF platform at the link https://osf.io/trk9g/overview?view_only=5d2c90061c474a61be8210ca9145fd91 (accessed on 24 December 2025).

### 2.2. Results

#### 2.2.1. Semantic Aspect of Narratives

**Figure 3:**
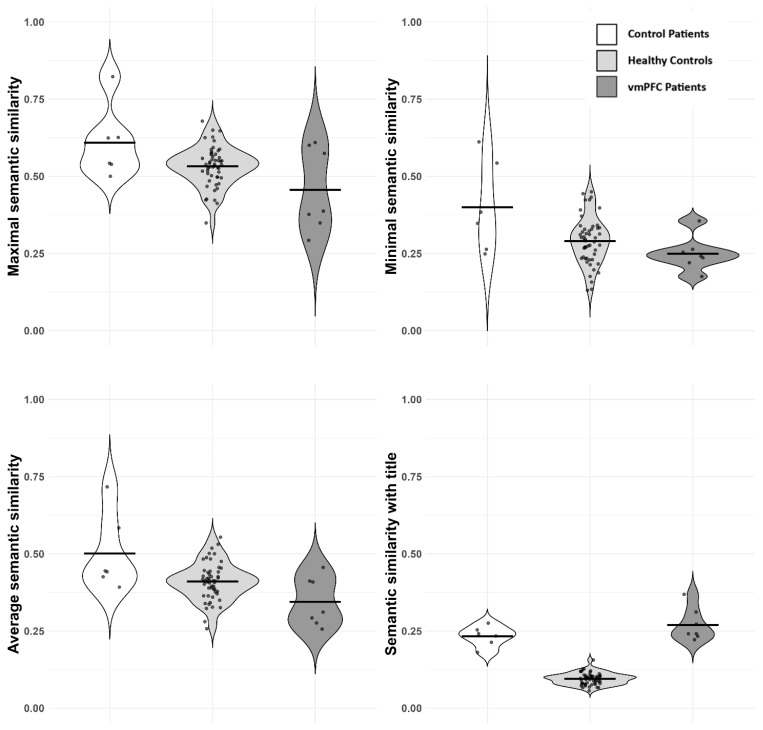
Results of semantic analyses (Exp. 1). From left to right: maximal point of semantic coherence, minimal point of semantic coherence (**top** panel), average semantic coherence, semantic coherence with the cue (**bottom** panel). Dots represent single participants; lines represent averages.

***Average semantic coherence***. There was no evidence for a correlation between the average number of sentences in the stories and their average semantic similarity (Pearson’s r = −0.07; BF_10_ = 0.32 ± 0%), so we conducted a one-way Bayesian ANOVA with the average local semantic coherence as the dependent variable and the variable group as the independent variable, finding evidence for group differences (BF_10_ = 25.3 ± 0%). Specifically, vmPFC patients displayed a lower local semantic coherence of text when compared to both healthy controls (0.35 vs. 0.41, BF.c = 56.3) and control patients (0.35 vs. 0.5, BF.c = 246), with control patients also demonstrating higher local semantic coherence than healthy controls (0.5 vs. 0.41, BF.c = 24.6; see Figure 3).

***Point of minimal semantic coherence***. There was strong evidence for a negative correlation between the length of stories and their point of minimal semantic coherence (r = −0.35; BF10 = 12.9 ± 0%), so here, we conducted a two-way Bayesian ANOVA with minimal semantic coherence as the dependent variable and the variables group and average length of stories as independent variables. The best model was the model that contained both the length of stories and the group as independent variables (BF_10_ = 91 ± 1.1%). The point of minimal coherence was indeed lower in vmPFC patients as compared to healthy controls (0.25 vs. 0.29; BF.c = 24) and control patients (0.25 vs. 0.4; BF.c = 106), with control patients also demonstrating higher minimal semantic coherence with respect to healthy controls (0.4 vs. 0.29; BF.c = 25.9).

***Point of maximal semantic coherence***. There was no evidence for a correlation between the length of stories and their point of maximal semantic coherence (r = 0.21; BF_10_ = 1 ± 0%). The one-way Bayesian ANOVA with the point of maximal semantic coherence as the dependent variable and the variable group as the independent variable revealed evidence for group differences (BF_10_ = 7.5 ± 0%), such that vmPFC patients had a lower point of maximal coherence (M = 0.46) when compared to healthy controls (M = 0.53; BF.c = 13.9) and control patients (M = 0.61; BF.c = 72.7), with control patients also demonstrating a higher point of maximal semantic coherence than healthy controls (BF.c = 16.6).

***Coherence of stories with their cue***. We computed the average semantic coherence of stories with their cue (the object’s name). There was extreme evidence for a negative correlation between the length of stories and the semantic coherence with their cue (r = −0.36; BF_10_ = 16 ± 0%), so we conducted a two-way Bayesian ANOVA with the average semantic coherence as the dependent variable, the variable group as the independent variable, and the average number of sentences as a covariate. The best model was the one containing only the group (BF_10_ = 3 × 10^24^ ± 0%). On average, vmPFC patients produced stories that were more thematically “anchored” to the cue with respect to the control groups (vmPFC vs. healthy: 0.27 vs. 0.1, BF.c = 2 × 10^13^; vmPFC vs. control patients: 0.27 vs. 0.23, BF.c = 14.7; healthy vs. control patients: 0.1 vs. 0.23; BF.c = 2 × 10^13^). Findings are confirmed with two other embedding models (E5-Multilingual and E5 ITA), and controlling for education levels; see Supplementary Materials.

***Global coherence***. There was no evidence for group differences in terms of global coherence (distance from the semantic centroid) (BF_10_ = 1.4 ± 0.01%), although vmPFC patients displayed a numerically lower average global coherence (M = 0.741) than healthy controls (M = 0.774) but not than control patients (M = 0.731).

#### 2.2.2. Linguistic Aspect of Narratives

***Pos Tagging***. There was evidence for group differences in the frequency of use of subordinating conjunctions (i.e., words that introduce a dependent, subordinate clause, linking it to a main clause, such as “that”, “because”, or “although”; BF_10_ = 29.7± 0.01%). In particular, vmPFC patients used more subordinating conjunctions than healthy controls (2.26% vs. 1.37%, BF.c = 107), with weak (anecdotal) evidence of differences between vmPFC patients and control patients (2.26% vs. 1.98%, BF.c = 2.5). On the other hand, the one-way Bayesian ANOVA on the normalized frequency of adjectives’ use revealed group differences (BF_10_ = 138 ± 0.01%) such that vmPFC patients were less prone to use adjectives in their discourse, relative to both control groups (vmPFC vs. healthy: 2.63% vs. 4.56%, BF.c = 6 × 10^5^; vmPFC vs. control patients: 2.63% vs. 3.14%, BF.c = 3; healthy vs. control patients: 4.56% vs. 3.14%, BF.c = 61). Furthermore, vmPFC patients used pronouns more frequently (BF_10_ = 8.5 ± 0.02%), both when compared to healthy controls (11.8% vs. 9%, BF.c = 539.5) and to control patients (11.8% vs. 10.6%, BF.c = 4.2). There was no evidence for differences in the other POS categories (all BF_10_ < 1.1).

***Lexical diversity—moving average type-token ratio (MATTR)***. There was evidence for group differences in terms of lexical diversity, as measured by the MATTR-25 scores (BF_10_ = 7.9 ± 0.01%), indicating that vmPFC patients’ lexicon was less varied than the two control groups (vmPFC patients, M = 0.894; healthy controls, M = 0.913; control patients, M = 0.906; vmPFC patients vs. healthy controls, BF.c = 19.8; vmPFC patients vs. control patients, BF.c = 5), with a superior lexical richness in healthy controls than in control patients (BF.c = 6.3). Results are replicated with a window of 15 tokens (MATTR-15; see Supplementary Materials).

## 3. Experiment 2: Event Cues

### 3.1. Methods

#### 3.1.1. Participants

We reanalyzed the dataset from Stendardi et al. [28], in which the sample included 8 patients with vmPFC lesions (6 M, mean age = 58 ± 5.9 years; mean education = 10.6 ± 3.2 years), 11 control patients with lesions outside the vmPFC (9 M, mean age = 55.8 ± 12.4; education = 13.3 ± 3.1), and 46 healthy participants (32 M, mean age = 58.2 ± 6.5; education = 11.5 ± 3.3). Participant groups were matched in age (BF_10_ = 0.2), education (BF_10_ = 0.6), and sex ratio (BF_10_ = 0.2). A total of 7 out of 8 vmPFC patients were involved in Exp. 1; one control patient was involved in Exp. 1. Participants provided informed consent to participate in the study, which was approved by the Bioethical Committee of the University of Bologna.

The background details of vmPFC patients and their neuropsychological scores have already been reported in Stendardi et al. [28]. We reprise the relevant data here: all vmPFC patients had lesions resulting from a ruptured aneurysm of the anterior communicating artery (AcoA; six bilateral, one right, one left). For control patients, lesion locations included the right temporal lobe (four patients), right fronto-temporal cortex (two patients), right temporo-parietal cortex, right occipito-temporal cortex, left temporal lobe, and left occipital cortex (one case each). All patients were in a stable chronic phase (>3 months post-injury). vmPFC patients displayed intact short-term memory (digit span performance, Equivalent Scores ≥ 1: mean = 3.1 ± 1.1; see [35]). Two vmPFC patients showed impaired long-term memory at the prose passage recall (Equivalent Score < 1; mean = 2.1 ± 1.6; see [35]), whilst the remaining six performed within the normal range. Six out of eight vmPFC patients demonstrated deficits in executive functioning, as measured by the Wisconsin Card Sorting Test (total number of errors Standard Score ≤ 81; mean = 77.6 ± 20.4; see [37]).

#### 3.1.2. Procedure

We analyzed data from the non-scripted condition (i.e., events that did not obey a strong underlying script), as it was the only condition where all participants were provided with the same 4 cues for event construction, and therefore allowed direct comparisons of the semantic aspect of stories in the three groups. Specifically, upon reading a sentence cue, participants were asked to imagine and describe out loud, in as much detail as possible, an event that could realistically happen to them in the future, while:

- Looking for their lost watch;
- Making friends in a new neighborhood;
- Entertain and baby-sit three children aged 4, 7 and 11 for a few hours;
- Retrieve a soccer ball that their niece/nephew threw in a tree.

All stories were audio-recorded and transcribed verbatim.

### 3.2. Results

#### 3.2.1. Semantic Aspect of Narratives

***Average semantic coherence***. Here, we found evidence for a correlation between the length of stories and their average semantic coherence (r = −0.46; BF_10_ = 123.9 ± 0%), so we conducted a two-way Bayesian ANOVA with the average semantic coherence as the dependent variable and the variable group and average number of sentences as the covariates. The best model was the model containing both independent variables (BF_10_ = 130.8 ± 0.8%), showing an additive effect of the two. Here, vmPFC patients displayed a higher local semantic coherence in their accounts compared to healthy controls (0.58 vs. 0.46; BF.c = 9.3), though there was not enough evidence for differences between vmPFC and control patients (0.58 vs. 0.53; BF.c = 2). Control patients displayed a higher local semantic coherence than healthy controls (0.53 vs. 0.46; BF.c = 16.3).

***Point of minimal semantic coherence***. There was again strong evidence for a negative correlation between the length of stories and their point of minimal semantic coherence (r = −0.6; BF_10_ = 2 × 10^4^ ± 0%), so we conducted the same two-way Bayesian ANOVA. The best model was the model that contained both the length of stories and the group as independent variables (BF_10_ = 3 × 10^4^ ± 0.6%). This time, the point of minimal coherence was higher in vmPFC patients with respect to healthy controls (0.52 vs. 0.31; BF.c = 38.8) and control patients (0.52 vs. 0.39; BF.c = 5.5), with control patients showing a higher minimal point of coherence with respect to healthy controls (0.39 vs. 0.31, BF.c = 5.8).

***Point of maximal semantic coherence***. There was again no evidence for a correlation between the length of stories and their point of maximal semantic coherence (r = 0.1; BF_10_ = 0.4 ± 0%), and there was no evidence for group differences (BF_10_ = 0.58 ± 0.01%).

**Figure 4:**
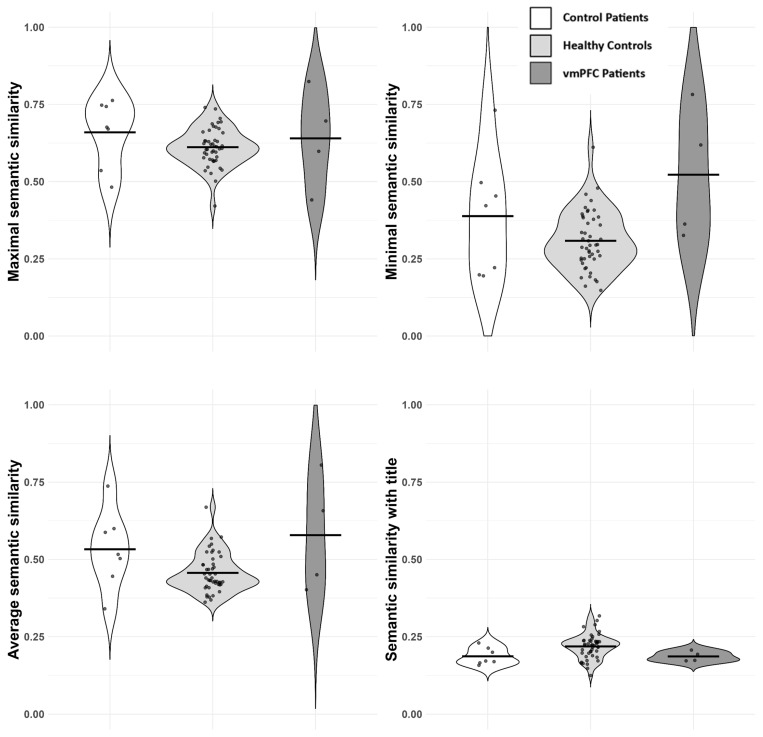
Results of semantic analyses (Exp. 2). From left to right: maximal point of semantic coherence, minimal point of semantic coherence (**top** panel), average of semantic coherence, semantic coherence with the cue (**bottom** panel). Dots represent single participants; lines represent averages.

***Coherence of stories with their cue***. There was no evidence for a correlation between the length of stories and the semantic coherence with their cue (r = 0.08; BF_10_ = 0.4 ± 0%), so we conducted a one-way Bayesian ANOVA with the average semantic coherence as the dependent variable and the variable group as the independent variable, finding no evidence for group differences (BF_10_ = 2.1 ± 0.01%). Results are represented in Figure 4. Findings are replicated (though only numerically) with two other embedding models (E5 Multilingual and E5 ITA) and are replicated exactly, controlling for education levels (see Supplementary Materials).

***Global coherence***. There was evidence for group differences in terms of global coherence (distance from the semantic centroid), even when controlling for the length of accounts (group + number of sentences, BF_10_ = 6.4 × 10^11^). vmPFC patients were, in fact, the highest in terms of global coherence (M = 0.839; healthy controls, M = 0.726; control patients, M = 0.802; vmPFC vs. healthy controls, BF.c = 698; vmPFC vs. control patients, BF.c = 3.8; control patients vs. healthy controls, BF.c = 370).

#### 3.2.2. Linguistic Aspect of Narratives

***Pos Tagging***. There was evidence for group differences in the normalized frequency of use of nouns, in that the two patient groups tended to fill the events with fewer word “entities” compared to healthy controls (e.g., “city”, “dog”, “clothes”; ANOVA BF_10_ = 1 × 10^7^; vmPFC patients: 11.6%; control patients: 11.7%; healthy controls: 16.9%; vmPFC vs. healthy, BF.c = 8 × 10^7^; control patients vs. healthy, BF.c = 2 × 10^13^). However, both patient groups used pronouns more frequently (ANOVA BF_10_ = 5.2) than healthy controls (vmPFC patients: 11.8%; control patients: 11.6%; healthy controls: 9.6%; vmPFC vs. healthy, BF.c = 13.3; control patients vs. healthy BF.c = 5 × 10^3^). Finally, as in Exp. 1, vmPFC patients tended to use fewer adjectives as compared to both control groups, with no differences between the control groups (ANOVA BF_10_ = 286 ± 0%; vmPFC patients: 2.4%; control patients: 3.1%; healthy controls: 4.4%; vmPFC vs. Healthy, BF.c = 1 × 10^6^; vmPFC patients vs. control patients, BF.c = 5.2). There was no evidence for differences in the other POS categories (all BF_10_ < 2).

***Lexical diversity—moving average type-token ratio (MATTR)***. There was no evidence for group differences in MATTR-25 scores (BF_10_ = 0.28 ± 0.02%), indicating that vmPFC patients varied their vocabulary as much as the control groups (vmPFC patients, M = 0.889 ± 0.01; healthy controls, M = 0.883; control patients, M = 0.888). Results are replicated with a 15-token window (see Supplementary Materials).

## 4. Discussion

The present study investigated the effects of vmPFC lesions on the semantic coherence and linguistic characteristics of spoken narratives, across two different experiments using object cues (Exp. 1) and sentence cues (Exp. 2).

Since previous evidence underscored a degradation of schematic knowledge following vmPFC damage [17,28,49,50], we expected vmPFC patients to show overall reduced semantic coherence in discourse. This prediction was only confirmed in Exp. 1, where participants had to imagine and describe an event starting from an object, but not in Exp. 2, where participants received a brief narrative prompt (sentence) as a cue.

Thus, our results are not consistent with the predicted generally reduced semantic coherence in vmPFC patients ([32]). Rather, they are suggestive of a more nuanced picture. In Exp. 1 (object cues), vmPFC patients showed reduced local semantic coherence compared to both healthy controls and control patients. This was evident across average, minimal, and maximal coherence measures. Moreover, their stories were more semantically tied to the object cue. Their global coherence of discourse was similar to the control groups, although lower than the healthy controls. At the linguistic level, vmPFC patients produced fewer adjectives, more pronouns, and displayed a reduced lexical diversity. On the contrary, in Exp. 2 (sentence cues), vmPFC patients displayed higher local semantic coherence than healthy controls (though similar to control patients). Also, they showed a higher point of minimal coherence and higher values of global coherence with respect to the two control groups. Further, vmPFC patients produced fewer nouns and adjectives, and more pronouns, but lexical diversity was not different in the three groups, indicating preserved overall vocabulary.

We propose a tentative interpretation of the apparently divergent findings in Exp. 1 and 2 that suggest that semantic coherence impairments in vmPFC patients depend on the nature of cues. Since vmPFC is deemed to drive the activation of schema-based knowledge for event construction [21,22], it is possible that, upon provision of a minimal (object) cue (e.g., balloon), vmPFC patients failed to activate detailed, relevant schemas (e.g., the typical birthday party) to then start the construction of a coherent event. The fact that vmPFC patients’ discourse remained abnormally anchored to the cue supports this interpretation. Accordingly, vmPFC patients are impaired at constructing events in response to single cue words ([5,6]). Extended (sentence) cues eschew the need for actual event construction because they provide an almost ready event on which patients only have to elaborate (see [51] for a discussion). This may have facilitated the activation of schemas. However, given that schemas are degraded and essentialized following vmPFC damage ([28]), patients’ constructed experience is bound to revolve around the core (major) actions of scripts, with a reduced possibility for creativity and the insertion of idiosyncratic content. This is consistent with a tendentially high semantic coherence of discourse, even when measured at the global level (i.e., mean distance from the semantic centroid).

On this view, the inconsistent impairment in semantic coherence of vmPFC patients might suggest that vmPFC damage affects multiple stages of schema processing and event construction, including both the initial triggering of event construction and its elaboration. Accordingly, vmPFC guides hippocampal activity to drive the unfolding of a complex event [21,52]. Moreover, vmPFC patients are impaired in constructing extended events and scenes ([5,26]), but can elaborate on moments selected from already constructed events ([27]) and from visually available scenes ([53]). Such findings are indeed supported by recent fMRI evidence highlighting vmPFC’s pivotal role in constructing temporally extended scenarios beyond single scenes and integrating low- and high-level mental representations to give rise to complex unfolding scenarios [24].

An alternative explanation is, of course, that vmPFC patients might not exhibit an inherent impairment in semantic coherence, and therefore, the apparent discrepancies across our two experiments may just reflect dataset-dependent effects. Accordingly, Kurczek & Duff [32] reported that bilateral vmPFC damage did not impair spoken discourse cohesion or coherence. It is important to note, however, that in Kurczek & Duff’s study, discourse was consistently elicited using extended cues (one Mediated Discourse Elicitation, MDEP [54], one picture description, and one story-retelling sample), akin to the cues we used in Exp. 2, and coherence was assessed through human judgment; as a result, this approach may have been less sensitive to potential inconsistencies across datasets. In this regard, it is important to draw a distinction between different operationalizations of semantic coherence. Human ratings capture the perceived flow of discourse, whereas linguistic computational metrics quantify the mathematical proximity of linguistic units (words, sentences) in a semantic space. Hence, the two metrics may provide insight on different levels of narrative organization: a story might be perceived as semantically coherent to a human listener while still exhibiting semantic drift or reduced semantic similarity when analyzed computationally, and vice versa, because the human listeners may infer meaning and coherence on the basis of their own life experiences, background knowledge, and idiosyncratic schemas. This distinction also explains why our approach captured subtle task-dependent fluctuations in coherence that were not apparent in the measures we had previously used and had not even caught our attention.

The interpretation we propose here also fits with our finding of a reduced lexical diversity in vmPFC patients’ stories in Exp. 1 but not 2, which is suggestive of a more pronounced impairment in generating events from minimal cues, even at the linguistic level. We note, however, two linguistic features characteristic of vmPFC patients’ discourse across Exp. 1 and Exp. 2: a higher frequency of pronouns, probably compensatory of the deficit in producing entities, and a lower frequency of adjectives. These linguistic features seem to be untied to the level of semantic coherence of the text, as they were evident in both experiments: one possibility is that increased pronoun use and reduced adjective use could reflect a consistent narrative style that favors referential continuity but limits the richness of description, which is in line with previous studies showing vmPFC patients’ impoverished episodic memory and future thinking tasks [4,5,6,7,26,55].

To conclude, we have shown that vmPFC damage affects semantic coherence and linguistic expression in a task-dependent manner: minimal cues (Exp. 1) lead to reduced local semantic coherence and lexical richness, with greater anchoring to the cue, likely reflecting impaired activation of schematic knowledge and event construction. Extended cues (Exp. 2), on the other hand, are capable of triggering schema activation and support the initiation of event construction, allowing vmPFC patients to at least depart from the cue. In this case, vmPFC patients remain nonetheless constrained to a limited semantic context, due to a failure in reinstating the finer aspects of schemas and their temporal structure ([28,56]), resulting in an enhanced local and global semantic coherence.

## Data Availability

All data (except for raw transcripts, for privacy concerns), are available in a publicly accessible repository at the link: https://osf.io/trk9g/overview?view_only=5d2c90061c474a61be8210ca9145fd91 (accessed on 24 December 2025).

## Supplementary Materials

The following supporting information can be downloaded at: https://www.mdpi.com/article/10.3390/e28050488/s1. References [43,44] are cited in the Supplementary Materials.
