# Supplementary material for: Contextual Modulation of Semantic Coherence in vmPFC Patients’ Mental Constructions
Source: Entropy (Basel). 2026 Apr 24;28(5):488. doi: 10.3390/e28050488 (PMC13205535; doi:10.3390/e28050488)
Supplement: Supplementary file 1 [file entropy-28-00488-s001.zip › entropy-4088606-supplementary.pdf]

# Supplementary Materials

## 1. Replication of analyses using two other embedding models (E5; intfloat/multilingual-e5-large, and E5 Ita; mik3ml/multilingual-e5-large-ita) from huggingface (see Wang et al., 2022)

*Note on the performance of the E5 Multilingual model: the globally higher coherence metrics obtained with E5 can be attributed to its retrieval-oriented training objective, which produces more compressed semantic representations that are less sensitive to subtle differences between utterances. This results in a distribution of similarities shifted toward higher values (Wang et al., 2022; 2024).*

**Exp. 1 - Object cues**

**E5 - Multilingual**

***Average semantic coherence***. There was no evidence for a correlation between the average number of sentences of stories and their average semantic similarity (Pearson's r = 0.07; $BF_{10}$ = 0.33 ± 0%), so we conducted a one-way Bayesian ANOVA with the average local semantic coherence as the dependent variable, and the variable group as the independent variable, finding evidence for group differences ($BF_{10}$ = 137 ± 0%). Specifically, vmPFC patients displayed a lower local semantic coherence of text when compared to both healthy controls (0.842 vs 0.863, BF.c = 566) and control patients (0.842 vs 0.856, BF.c = 7.5), with control patients also demonstrating a slightly lower local semantic coherence than healthy controls (0.856 vs 0.863, BF.c = 3.7).

***Point of minimal semantic coherence.*** There was evidence for a negative correlation between the length of stories and their point of minimal semantic coherence (r = -0.29; BF10 = 3.4 ±0%), so here we conducted a two-way Bayesian ANOVA with minimal semantic coherence as the dependent variable, and the variables group and average length of stories as independent variables. The best model was the model that contained both the length of stories and the group as independent variables ($BF_{10}$ = 80 ±1.1%). The point of minimal coherence was indeed lower in vmPFC patients as compared to healthy controls (0.824 vs 0.838; BF.c = 849) and control patients (0.824 vs 0.835; BF.c = 6.8) with no differences between the control groups (BF.c = 1.9).

***Point of maximal semantic coherence***. There was evidence for a correlation between the length of stories and their point of maximal semantic coherence (r = 0.32; $BF_{10}$ = 6.3 ± 0%). The two-way Bayesian ANOVA with the point of maximal semantic coherence as the dependent variable, and the variable group as the independent variable, with number of sentences as covariate revealed evidence for group differences ($BF_{10}$ Group + number of sentences = 28 ± 2.68%), such that vmPFC patients had a lower point of maximal coherence (M = 0.864) when compared to healthy controls (M = 0.887; BF.c = 29.3) and control patients (M = 0.878; BF.c = 3.9), with control patients also demonstrating a higher point of maximal semantic coherence than healthy controls (BF.c = 3.5).

***Coherence of stories with their cue***. There was extreme evidence for a negative correlation between the length of stories and the semantic coherence with their cue (r = -0.46; $BF_{10}$ = 256 ± 0%), so we conducted a two-way Bayesian ANOVA with the average semantic coherence as the dependent variable, the variable group as the independent variable, and the average number of sentences as a covariate. The best model was the one containing both variables ($BF_{10}$ = $1.7*10^{14}$ ± 1.19%), meaning they both influenced the semantic similarity with the cue. On average, vmPFC patients produced stories that were more thematically "anchored" to the cue with respect to the control groups, confirming our main findings (vmPFC vs Healthy: 0.791 vs 0.765, BF.c = $4*10^{8}$; vmPFC vs Control Patients: 0.791 vs 0.782, BF.c = 18; Healthy vs Control Patients: 0.765 vs 0.782; BF.c = $2*10^{6}$).

**E5 - ITA**

***Average semantic coherence***. There was no evidence for a correlation between the average number of sentences of stories and their average semantic similarity (Pearson's r = 0.07; $BF_{10}$ = 0.3 ± 0%), so we conducted a one-way Bayesian ANOVA with the average local semantic coherence as the dependent variable, and the variable group as the independent variable, finding evidence for group differences ($BF_{10}$ = 23.7 ± 0%). Specifically, vmPFC patients displayed a lower local semantic coherence of text when compared to both healthy controls (0.497 vs 0.576, BF.c = 232) and control patients (0.497 vs 0.593, BF.c = 56), with no differences between control patients and healthy controls (0.593 vs 0.576, BF.c = 2.2).

***Point of minimal semantic coherence.*** There was evidence for a negative correlation between the length of stories and their point of minimal semantic coherence (r = -0.34; $BF_{10}$ = 9.5 ±0%), so here we conducted a two-way Bayesian ANOVA with minimal semantic coherence as the dependent variable, and the variables group and average length of stories as independent variables. The best model was the model that contained both the length of stories and the group as independent variables ($BF_{10}$ = 14.8 ±0.7%). The point of minimal coherence was indeed lower in vmPFC patients as compared to healthy controls (0.426 vs 0.464; BF.c = 16.3) and control patients (0.426 vs 0.509; BF.c = 17.1) with control patients being slightly higher than healthy controls (0.509 vs 0.464; BF.c = 4.8).

***Point of maximal semantic coherence***. There was evidence for a correlation between the length of stories and their point of maximal semantic coherence (r = 0.42; $BF_{10}$ = 43 ± 0%). The two-way Bayesian ANOVA with the point of maximal semantic coherence as the dependent variable, and the variable group as the independent variable, with number of sentences as covariate revealed evidence for group differences ($BF_{10}$ Group + number of sentences = 499.6 ± 0.7%), such that vmPFC patients had a lower point of maximal coherence (M = 0.577) when compared to healthy controls (M = 0.683; BF.c = 84) and control patients (M = 0.667; BF.c = 14.6), with no differences between control patients and healthy controls (BF.c = 1.9).

***Coherence of stories with their cue***. There was not enough evidence for a negative correlation between the length of stories and the semantic coherence with their cue (r = -0.23; $BF_{10}$ = 1.3 ± 0%), so we conducted a one-way Bayesian ANOVA with the average semantic coherence as the dependent variable and the variable group as the independent variable, which revealed group differences ($BF_{10}$ = 15.4 ± 0%). On average, vmPFC patients produced stories that were more thematically "anchored" to the cue with respect to the control groups, again confirming our findings (vmPFC vs Healthy: 0.381 vs 0.341, BF.c = 29.2; vmPFC vs Control Patients: 0.381 vs 0.328, BF.c = 49.5; Healthy vs Control Patients: 0.341 vs 0.328; BF.c = 4.5).

**Exp. 2 - Event cues**

**E5 - Multilingual**

***Average semantic coherence***. Here, we did not find enough evidence for a correlation between the length of stories and their average semantic coherence (r = -0.27; $BF_{10}$ = 1.9 0%), so we conducted a one-way Bayesian ANOVA with the average semantic coherence as the dependent variable, and the variable group as predictor, finding no effect of group ($BF_{10}$ = 0.44). However, on average, vmPFC patients displayed higher semantic coherence (M = 0.871) than the control groups (Healthy, M = 0.859; Control patients, M = 0.857).

***Point of minimal semantic coherence***. There was strong evidence for a negative correlation between the length of stories and their point of minimal semantic coherence (r = -0.63; $BF_{10}$ = $7*10^4$ ±0%), so we conducted the same two-way Bayesian ANOVA. The best model was the model that only contained the length of stories ($BF_{10}$

= $6*10^4 \pm 0.01\%$). Again, the point of minimal coherence was higher in vmPFC patients (0.853) than in the control groups (Healthy, M = 0.824; Control patients, M = 0.835).

***Point of maximal semantic coherence***. There was evidence for a correlation between the length of stories and their point of maximal semantic coherence (r = 0.37; $BF_{10} = 11.1 \pm 0\%$), and there was no evidence for group differences (best model, number of sentences, $BF_{10} = 8.4 \pm 0\%$).

***Coherence of stories with their cue***. There was no evidence for a correlation between the length of stories and the semantic coherence with their cue (r = 0.06; $BF_{10} = 0.3 \pm 0\%$), so we conducted a one-way Bayesian ANOVA with the average semantic coherence as the dependent variable, and the variable group as the independent variable, finding no evidence for group differences ($BF_{10} = 0.59 \pm 0.02\%$).

**E5 - ITA**

***Average semantic coherence***. Here, we found evidence for a negative correlation between the length of stories and their average semantic coherence (r = -0.38; $BF_{10} = 14.5 \pm 0\%$), so we conducted a two-way Bayesian ANOVA with the average semantic coherence as the dependent variable, and the variable group and average number of sentences as the covariate. The best model was the model containing only the average number of sentences ($BF_{10} = 10.9 \pm 0\%$). Nonetheless, vmPFC patients displayed a higher local semantic coherence in their accounts (vmPFC, M = 0.663; Healthy, M = 0.598; Control patients, M = 0.596).

***Point of minimal semantic coherence***. There was again strong evidence for a negative correlation between the length of stories and their point of minimal semantic coherence (r = -0.67; $BF_{10} = 2*10^5 \pm 0\%$), so we conducted a two-way Bayesian ANOVA. The best model was the model that contained only the length of stories ($BF_{10} = 6*10^5 \pm 0.01\%$). Again, the point of minimal coherence was higher in vmPFC patients (M = 0.601) with respect to healthy controls (M = 0.459) and control patients (M = 0.506).

***Point of maximal semantic coherence***. There was again no evidence for a correlation between the length of stories and their point of maximal semantic coherence (r = 0.26; $BF_{10} = 1.6 \pm 0\%$), and there was no evidence for group differences ($BF_{10} = 0.48 \pm 0.01\%$).

***Coherence of stories with their cue*** There was no evidence for a correlation between the length of stories and the semantic coherence with their cue (r = 0.04; $BF_{10} = 0.3 \pm 0\%$), so we conducted a one-way Bayesian ANOVA with the average semantic coherence as the dependent variable, and the variable group as the independent variable, finding no evidence for group differences ($BF_{10} = 0.34 \pm 0.03\%$).

## 2. Excerpts with low and high coherence from one healthy participant in Exp.1, and comparative performance on cosine similarity computation between adjacent sentences of the three embedding models

# Low coherence

### Example 1

"Ci sono degli spazi adibiti per i vari pescatori, che sono tutti corredati cioè attrezzati di sgabelli per sedersi e anche ombrelloni non troppo grandi, e c'è chi ha anche più persone intorno, magari che si mettono su una coperta da pic-nic o qualcosa del genere. / Il tempo è buono e si sta bene."

(*There are designated spots for the various fishermen, all equipped with stools to sit on and also with not-too-large umbrellas, and some even have more people around, perhaps sitting on a picnic blanket or something like that. / The weather is nice, and it's comfortable*.)

| Model | Local coherence |
|---|---|
| S-BERT | 0.1473 |
| E5 | 0.8083 |
| E5-ITA | 0.4083 |

**Example 2**

"E' una giornata un po' nuvolosa, e non c'è tanta gente. / Mi dirigo nel supermercato, quindi prendo il carrello, ed entro dentro per fare la spesa."
(*It's a slightly cloudy day, and there aren't many people around. / I head into the supermarket, grab a shopping cart, and go in to do the shopping*.)

| Model | Local coherence |
|---|---|
| S-BERT | 0.1243 |
| E5 | 0.8305 |
| E5-ITA | 0.4803 |

## High coherence

**Example 1**

"Non so che caspita sia successo davanti, ma sono tutti fermi, per cui ho lo specchietto retrovisore davanti e ogni tanto lo guardo, sperando che non mi venga addosso nessuno. / Guardo anche gli specchietti laterali."
(*I have no idea what the heck happened up ahead, but everyone is stopped, so I keep an eye on the rearview mirror and glance at it every now and then, hoping no one will crash into me. / I also check the side mirrors.)*

| Model | Local coherence |
|---|---|
| S-BERT | 0.5291 |
| E5 | 0.8754 |
| E5-ITA | 0.7254 |

**Example 2**

"C'è un tavolo con un vassoio con tante caramelle colorate, e ci sono dei pasticcini, pop-corn, patatine e varie bibite, tutti i bambini che giocano all'aperto e si divertono. / Ogni tanto, qualcuno va a prendere qualcosa al tavolo.
*(There's a table with a tray full of colorful candies, and there are also pastries, popcorn, chips, and various drinks, all the children are playing outside and having fun. / Every now and then, someone goes over to grab something from the table.)*

| Model | Local coherence |
|---|---|
| S-BERT | 0.526 |
| E5 | 0.8327 |
| E5-ITA | 0.5355 |

## 3. Confirmation of analyses controlling for education levels

### Exp. 1 – Object cues

| Dependent variable | Correlation with years of education | Correlation with presence of secondary education (yes/no) | Replication of main analysis with years of education as covariate | Replication of main analysis with secondary education (yes/no) as covariate |
|---|---|---|---|---|
| Average semantic coherence | No (r = - 0.1; $BF_{10}$ = 0.4) | No (r = - 0.07; $BF_{10}$ = 0.3) | Yes (Best model: Group, $BF_{10}$ = 25.3) | Yes (Best model: Group, $BF_{10}$ = 25.3) |
| Point of minimal semantic coherence | No (r = - 0.1; $BF_{10}$ = 0.39) | No (r = - 0.13; $BF_{10}$ = 0.47) | Yes (Best model: Group, $BF_{10}$ = 7.3) | Yes (Best model: Group, $BF_{10}$ = 7.3) |
| Point of maximal semantic coherence | No (r = 0.005; $BF_{10}$ = 0.28) | No (r = - 0.04; $BF_{10}$ = 0.3) | Yes (Best model: Group, $BF_{10}$ = 7.5) | Yes (Best model: Group, $BF_{10}$ = 7.5) |
| Semantic coherence with cue | No (r = - 0.25; $BF_{10}$ = 1.8) | No (r = - 0.16; $BF_{10}$ = 0.58) | Yes (Best model: Group + Secondary Education, $BF_{10}$ = $2*10^{21}$) | Yes (Best model: Group + Secondary Education, $BF_{10}$ = $2.5*10^{22}$) |
| Global coherence | No (r = - 0.25; $BF_{10}$ = 1.8) | No (r = - 0.24; $BF_{10}$ = 1.5) | Yes (Best model: Group + Secondary Education, $BF_{10}$ = 4.2) | Yes (Best model: Group + Secondary Education, $BF_{10}$ = 3.2) |

Even though our original samples were statistically matched for age, education, and sex ratio, we also subsampled the two control groups to obtain a closer match in terms of age, sex, and education, and re-ran our analyses. We retained 34 healthy controls, 4 control patients and 4 vmPFC patients.

| Group | Age | Education | Sex | Average Semantic coherence | Minimal Semantic coherence | Maximal Semantic coherence | Coherence with cue | Global coherence |
|---|---|---|---|---|---|---|---|---|
| **Control patients** | 57.2 | 14 | 2M,2F | 0.534 | 0.451 | 0.623 | 0.228 | 0.793 |
| **Healthy controls** | 58.3 | 13.9 | 21M, 13F | 0.407 | 0.282 | 0.535 | 0.095 | 0.725 |
| **vmPFC patients** | 57.8 | 13.2 | 3M, 1F | 0.364 | 0.259 | 0.487 | 0.242 | 0.727 |

Even with smaller and more closely matched samples, we confirmed our analyses on average coherence ($BF_{10}$ Group = 15.5 ±0%), point of minimal coherence ($BF_{10}$ Group = 37.9 ±0%), and coherence with the story's cue ($BF_{10}$ Group = $3.4*10^{11}$ ±0%). The effect of global coherence appears, indicating that healthy controls are the most globally coherent ($BF_{10}$ Group = 4.7 ±0.01%). The effect is reduced for the analysis on the point of

maximal coherence, but vmPFC patients still display a lower point of maximal coherence with respect to the other groups ($BF_{10}$ = 1.8).

### Exp. 2 – Event cues

| Dependent variable | Correlation with years of education | Correlation with presence of secondary education (yes/no) | Replication of main analysis with years of education as covariate | Replication of main analysis with secondary education (yes/no) as covariate |
|---|---|---|---|---|
| Average semantic coherence | No (r = 0.06; $BF_{10}$ = 0.3) | No (r = 0.12; $BF_{10}$ = 0.4) | Yes (Best model: Group, $BF_{10}$ = 11.5) | Yes (Best model: Group, $BF_{10}$ = 11.5) |
| Point of minimal semantic coherence | No (r = - 0.08; $BF_{10}$ = 0.4) | No (r = - 0.03; $BF_{10}$ = 0.3) | Yes (Best model: Group, $BF_{10}$ = 15.2) | Yes (Best model: Group, $BF_{10}$ = 15.2) |
| Point of maximal semantic coherence | Yes (r = 0.3; $BF_{10}$ = 3.6) | Yes (r = 0.34; $BF_{10}$ = 6.9) | Yes (Best model: Years of education, $BF_{10}$ = 2.8) | Yes (Best model: Secondary education, $BF_{10}$ = 5.3). |
| Semantic coherence with cue | Anecdotal evidence (r = 0.29; $BF_{10}$ = 2.8) | No (r = 0.22; $BF_{10}$ = 1) | Yes (Best model: Years of education, $BF_{10}$ = 2.2) | Yes (Best model: Secondary education, $BF_{10}$ = 0.9) |
| Global coherence | No (r = - 0.12; $BF_{10}$ = 0.4) | No (r = - 0.11; $BF_{10}$ = 0.4) | Yes (Best model: Group, $BF_{10}$ = 340) | Yes (Best model: Group, $BF_{10}$ = 340) |

*(Note that for the point of maximal coherence and the coherence with the cue, which are the only dependent variables correlating with education levels, there was no effect of the group in the original analyses; such effect is also absent in the current analyses, replicating the original findings).*

Even though our original samples were statistically matched for age, education, and sex ratio, we again repeated our analyses subsampling the two control groups to obtain a closer match to vmPFC patients in terms of age, and education. Given the relatively small sample size of our patients' groups, it was not possible to match them on sex distribution. We retained 22 healthy controls, 4 control patients and 4 vmPFC patients.

| Group | Age | Education | Sex | Average Semantic coherence | Minimal Semantic coherence | Maximal Semantic coherence | Coherence with cue | Global coherence |
|---|---|---|---|---|---|---|---|---|
| **Control Patients** | 56.5 | 11.0 | 4M, 0F | 0.472 | 0.335 | 0.591 | 0.190 | 0.786 |
| **Healthy** | 56.1 | 10.4 | 15M, 7F | 0.449 | 0.306 | 0.595 | 0.217 | 0.728 |
| **vmPFC** | 57.2 | 10.3 | 2M, 2F | 0.579 | 0.522 | 0.640 | 0.197 | 0.839 |

Even with a smaller sample size closely matched in age and education, we replicated exactly our analyses on average coherence ($BF_{10}$ Group = 5.9 ±0%), point of minimal coherence ($BF_{10}$ Group = 19.2 ±0%), coherence

with the story's cue ($BF_{10}$ Group = $3.4*10^{11}$ ±0%), and global coherence ($BF_{10}$ Group = 84.3 ±0%). The effect of the Group was (and still is) absent for the analyses on the maximal point of semantic coherence and coherence with the cue (all $BF_{10} < 0.6$).

## 4. Qualitative comparison between experiments

We compared the performance of three vmPFC patients for which we have valid data in both experiments. Remarkably, the minimal point of coherence is lower in Exp.1 than in Exp 2. for all three patients, and the average semantic coherence is again lower in Exp. 1 than in Exp. 2 for two patients (for the other patient, the average is almost the same). The semantic attachment to the cue is higher in Exp. 1 than in Exp. 2 for two out of three patients. Finally, for all patients, global coherence is higher in Exp. 2 (event cues).

| Patient | Minimal coherence Exp.1 | Minimal coherence Exp.2 | Average coherence Exp.1 | Average coherence Exp.2 | Semantic similarity with cue Exp.1 | Semantic similarity with cue Exp.2 | Global coherence Exp. 1 | Global coherence Exp. 2 |
|---|---|---|---|---|---|---|---|---|
| P2 | 0.25 | 0.36 | 0.41 | 0.40 | 0.24 | 0.17 | 0.73 | 0.82 |
| P4 | 0.26 | 0.78 | 0.41 | 0.80 | 0.19 | 0.23 | 0.69 | 0.92 |
| P8 | 0.18 | 0.33 | 0.28 | 0.45 | 0.24 | 0.17 | 0.72 | 0.75 |

## 5. Replication of analysis on lexical diversity with MATTR-15:

**Exp. 1 (Object cues):**

There was evidence for group differences in terms of lexical diversity, as measured by the MATTR-15 scores ($BF_{10}$ = 10.6 ± 0.01%), indicating that vmPFC patients' lexicon was less varied than healthy controls but not control patients (vmPFC patients, M = 0.932; healthy controls, M = 0.945; control patients, M = 0.938; vmPFC patients vs healthy controls, BF.c = 22.5; vmPFC patients vs control patients, BF.c = 1.5), with a superior lexical richness in healthy controls than in control patients (BF.c = 22.7).

**Exp. 2 (Event cues):**

There was no evidence for group differences in MATTR-15 scores ($BF_{10}$ = 0.34 ± 0.03%), indicating that vmPFC patients varied their vocabulary as much as the control groups (vmPFC patients, M = 0.932; healthy controls, M = 0.924; control patients, M = 0.930).

## 6 Supplementary references

Wang, L., Yang, N., Huang, X., Jiao, B., Yang, L., Jiang, D., Majumder, R & Wei, F., (2022). Text embeddings by weakly-supervised contrastive pre-training. arXiv preprint arXiv:2212.03533).

Wang, L., Yang, N., Huang, X., Yang, L., Majumder, R., & Wei, F. (2024). Multilingual e5 text embeddings: A technical report. arXiv preprint arXiv:2402.05672.
